# Block of TREK and TRESK K2P channels by lamotrigine and two derivatives sipatrigine and CEN-092

**DOI:** 10.1016/j.bbrep.2021.101021

**Published:** 2021-05-19

**Authors:** Yvonne Walsh, Michael Leach, Emma L. Veale, Alistair Mathie

**Affiliations:** a Medway School of Pharmacy, University of Kent and University of Greenwich, Central Avenue, Chatham Maritime, ME4 4TB, UK; b University of Greenwich, Central Avenue, Chatham Maritime, ME4 4TB, UK; c School of Engineering, Arts, Science and Technology, University of Suffolk, Waterfront Building, Neptune Quay, Ipswich, IP4 1QJ, UK

**Keywords:** TREK channel, TRESK channel, K2P channel, Lamotrigine, Sipatrigine, CEN-092

## Abstract

TREK and TRESK K2P channels are widely expressed in the nervous system, particularly in sensory neurons, where they regulate neuronal excitability. In this study, using whole-cell patch-clamp electrophysiology, we characterise the inhibitory effect of the anticonvulsant lamotrigine and two derivatives, sipatrigine and 3,5-diamino-6-(3,5-bistrifluoromethylphenyl)-1,2,4-triazine (CEN-092) on these channels.

Sipatrigine was found to be a more effective inhibitor than lamotrigine of TREK-1, TREK-2 and TRESK channels. Sipatrigine was slightly more potent on TREK-1 channels (EC_50_ = 16 μM) than TRESK (EC_50_ = 34 μM) whereas lamotrigine was equally effective on TREK-1 and TRESK. Sipatrigine was less effective on a short isoform of TREK-2, suggesting the N terminus of the channel is important for both inhibition and subsequent over-recovery. Inhibition of TREK-1 and TREK-2 channels by sipatrigine was reduced by mutation of a leucine residue associated with the norfluoxetine binding site on these channels (L289A and L320A on TREK-1 and TREK-2, respectively) but these did not affect inhibition by lamotrigine. Inhibition of TRESK by sipatrigine and lamotrigine was attenuated by mutation of bulky phenylalanine residues (F145A and F352A) in the inner pore helix. However, phosphorylation mutations did not alter the effect of sipatrigine. CEN-092 was a more effective inhibitor of TRESK channels than TREK-1 channels.

It is concluded that lamotrigine, sipatrigine and CEN-092 are all inhibitors of TREK and TRESK channels but do not greatly discriminate between them. The actions of these compounds may contribute to their current and potential use in the treatment of pain and depression.

## Highlights

• Lamotrigine blocks TREK and TRESK potassium channels at clinical concentrations.
• Sipatrigine is more effective than lamotrigine at blocking TREK and TRESK channels.
• Mutation of norfluoxetine binding site on TREK channels attenuates sipatrigine block.
• Residues in the inner pore region of TRESK channels regulate sipatrigine block.
• The novel lamotrigine derivative, CEN-092, blocks TRESK channels.

## 1 Introduction

The TWIK-related potassium channels (TREK-1 and TREK-2) and TWIK-related spinal cord potassium channel (TRESK) belong to the two-pore domain (K2P) family of ion channels, whose main functional role is to regulate cellular excitability [1]. TREK-1 and TREK-2 are highly expressed in small nociceptor dorsal root ganglion (DRG) and have been shown to play an active role in neuroprotection, schizophrenia, depression, and pain, whilst TRESK, with similar high expression in sensory neurons, has a role in nociception and migraine [[1], [2], [3], [4]]. Compounds which alter the activity of these channels are therefore predicted to have therapeutic potential in treating CNS disorders. Indeed, TREK-1 and TREK-2, channel activity has been shown to be regulated by antidepressants, antipsychotics, mood stabilizers, anaesthetics and neuroprotective agents [[4], [5], [6], [7], [8], [9]]. TRESK channels have been shown to be regulated by volatile anaesthetics [10], anti-depressant and anti-convulsant agents [11] and antihistamines [12].

Sipatrigine (BW 619C89) is a known neuroprotective agent derived from the clinically used antiepileptic agent lamotrigine [13]. Sipatrigine has previously been shown to be a potent antagonist of TREK-1, and to a lesser extent, TRAAK channels [5,14]. Lamotrigine also modulates neuronal activity and is used both as an anti-convulsant agent and in the treatment of bipolar disorder [15]. Lamotrigine has previously been shown to be an antagonist of TRESK channels [11] with little effect on TREK-1 or TRAAK currents [5]. As a result, lamotrigine has been used to distinguish TRESK channels from other channels in functional experiments [10,16].

More recent advances have revealed possible mechanisms by which some modulators of K2P channels activity may act. The co-crystallised structure of TREK-2 with norfluoxetine has shown multiple activation states with distant fenestration regions and key “contact points” within them [17]. Furthermore, molecular docking simulations and homology modelling has identified two key residues in TRESK channels, involved in binding blockers of K channels, such as propafenone and lidocaine [18]. Alanine mutations of the identified amino acids in TRESK channels diminished inhibition by the TRESK blockers quinine, propafenone, lidocaine and aristolochic acid [12,19].

In this study, we determine the relative pharmacological effects of sipatrigine and lamotrigine on human TRESK, TREK-1 and TREK-2 channels. We further characterise the effect of these compounds utilizing reported binding site mutations to try to gain insight into their binding and/or mechanism of action. Additionally, for the first time, we describe and characterise the effect of a novel lamotrigine-derived compound, 3,5-diamino-6-(3,5-bistrifluoromethylphenyl)-1,2,4-triazine (CEN-092) on TREK-1 and TRESK channels.

## 2 Materials and Methods

Most of the methods used here have been described previously [20,21] and a detailed explanation can be found in Ref. [22]. A summary of the methods used and any variations specific to this study are given below.

### 2.1 Mammalian expression plasmids

Human TREK-1 (KCNK2, Genbank™ NP_055032.1) and TREK2 (KCNK10, NP_612190.1) was cloned into pcDNA3.1^+^ (Invitrogen, Carlsbad, CA, USA) and PCMV6-XL-4 (OriGene Technologies, Inc. USA). Human TRESK (KCNK18, NP_862823.1) was cloned into pFAW-Ac-GFP vector and was a kind gift from Dr Eddie Stevens, Pfizer, UK.

### 2.2 Mutations

Point mutations were introduced into TREK-1, TREK-2 and TRESK by site-directed mutagenesis using the QuikChange (Agilent, CA, USA) method as previously described [20]. All constructs were fully sequenced to ensure correct mutation incorporation (Eurofins MWG Operon, Ebersberg, Germany). To generate the N-terminally truncated alternative translation initiation (ATI) isoforms of TREK-2: short (TREK-2_Δ1-63; 471 amino acids (AA)) and intermediate (TREK-2_Δ1-63_M72I; 483 AA) a deletion and site-directed mutagenesis strategy (Quikchange, Agilent, CA, USA) was utilised. For the forced full-length TREK-2 construct (543 AA), methionine 60 (M60) and M72 were mutated to isoleucine (I).

### 2.3 In silico channel mutation

The crystal structure of hTREK-1 (PDB ID: 4TWK) was visualised using PyMol v1.7 and Modeller v9.18. To create the mutation in the model, a residue mutation model script was used [23].

### 2.4 Cell culture

All experiments were performed using a modified human embryonic kidney 293 cell line, tsA201 (European Collection of Authenticated Cell Cultures; Sigma-Aldrich, UK), prepared and maintained as previously described [20].

### 2.5 Transfection

All WT and mutant constructs, including a green fluorescent protein (GFP)-expressing vector, were transiently transfected at a concentration of 0.5 μg, into tsA201 cells, using a modified calcium-phosphate transfection method [20,22].

### 2.6 Whole-cell patch-clamp electrophysiology

Recordings were made from GFP-fluorescing tsA201 cells, expressing the protein of interest using whole-cell patch-clamp in a voltage clamp configuration. Whole–cell currents were recorded from cells maintained at a holding potential of −60 mV at 20–24 °C (room temperature). A combined voltage-step and voltage-ramp protocol was applied using an Axopatch 200B patch-clamp amplifier (Molecular Devices, Sunnyvale, CA). Cells were first hyperpolarized to −80 mV for 100 ms and then stepped to −40 mV for 500 ms, then stepped to −120 mV for 100 ms (the “voltage-step” component). This was followed by a 500-ms ramp change in voltage to +20 mV and a step back to −80 mV for another 100 ms before being returned to the holding potential of −60 mV (the “voltage-ramp” component). This protocol was composed of sweeps lasting 1.5 s (s), including sampling at the holding voltage and was repeated once every 5 s. For all experiments, the external recording solution comprised of 145 mM NaCl, 2.5 mM KCl, 3 mM MgCl_2_, 1 mM CaCl_2_ and 10 mM HEPES (pH to 7.4, using NaOH). The internal recoding solution contained 150 mM KCl, 3 mM MgCl_2_, 5 mM EGTA and 10 mM HEPES (pH adjusted to 7.4 with KOH).

### 2.7 Compounds

Sipatrigine and lamotrigine were purchased from Tocris. Cen-092 was supplied by Dr Mike Leach (University of Greenwich). The compound stock solutions were made up in 100% DMSO or ethanol.

### 2.8 Data analysis and statistics

Current obtained was analysed using pClamp 10.2 software (Molecular Devices). Quantification of compound effects was carried out using currents obtained from the voltage-step component of the protocol. Any further analysis and statistical tests were performed using GraphPad Prism v8.0.1 (Graphpad, USA). Data are expressed as mean values with a range of 95% Confidence Intervals (CI) and *n* represents the number of individual cells. Statistical comparisons were made using unpaired two-tailed Welch's t-tests and one-way ANOVA tests with a post hoc Dunnett's or Tukey's method for multiple comparison test. P < 0.05 was considered statistically significant.

## 3 Results & discussion

### 3.1 Sipatrigine is a more effective inhibitor than lamotrigine of both TREK and TRESK channels

**Fig. 1:**
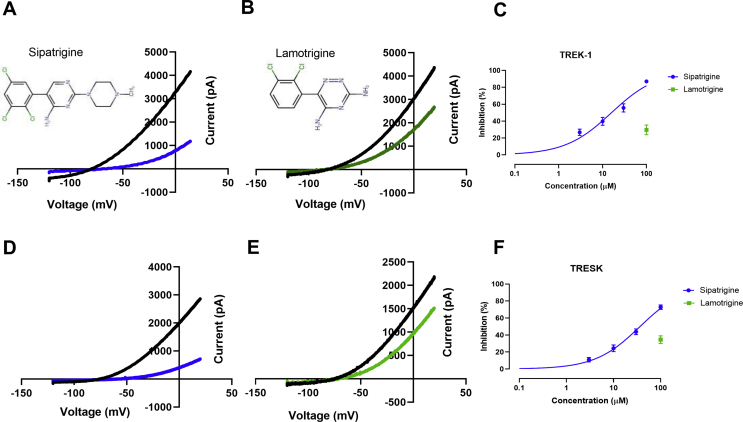
Inhibition of TREK-1 and TRESK channels by sipatrigine (blue) and lamotrigine (green). (A) Current-voltage relationship for TREK-1 in the presence and absence of sipatrigine (100 μM) obtained using the voltage-ramp protocol. Insert shows chemical structure of sipatrigine. (B) Current-voltage relationship for TREK-1 in the presence and absence of lamotrigine (100 μM). Insert shows chemical structure of lamotrigine. (C) Concentration-response curve for sipatrigine inhibition of TREK-1 current. (D) Current-voltage relationship for TRESK in the presence and absence of sipatrigine (100 μM). (E) Current-voltage relationship for TRESK in the presence and absence of lamotrigine (100 μM). (F) Concentration-response curve for sipatrigine inhibition of TRESK current. (For interpretation of the references to colour in this figure legend, the reader is referred to the Web version of this article.)

Our initial experiments sought to determine the pharmacological profile of sipatrigine and lamotrigine on TRESK and TREK-1 channels to determine if these compounds did indeed show selectivity between channels. Utilizing cloned human wildtype (WT) TREK-1 and TRESK channels, transiently expressed in tsA201 cells, whole-cell current was measured using patch-clamp electrophysiology in the absence and presence of sipatrigine and lamotrigine (see Materials and Methods). Acute application of sipatrigine (100 μM) resulted in a potent and reversible inhibition of TREK-1 by 87% [95% confidence interval: 85–92; n = 19, Fig. 1A]. Lamotrigine, at the same concentration, had a smaller 30% inhibitory effect on TREK-1 channels [95% CI: 11–53; n = 6, Fig. 1B]. Sipatrigine had a 50% effective concentration (EC_50_) of 16 μM [95% CI: 12–20] and a Hill slope of 0.82 [95% CI: 0.7–1, Fig. 1C].

Sipatrigine (100 μM) caused a substantial, 73%, inhibition of TRESK channels [95% CI: 69–82; n = 16, Fig. 1D] whilst the same concentration of lamotrigine inhibited TRESK channels by 35% [95% CI: 9–48; n = 8, Fig. 1E]. The inhibitory effect of sipatrigine was significantly less on TRESK (p < 0.05 [95% CI -17 to −8]) than was observed for TREK-1, with sipatrigine having an EC_50_ of 34 μM [95% CI: 29–39] and a Hill slope of 0.95 [95% CI: 0.8–1, Fig. 1F] on the former.

Sipatrigine (100 μM) produced a potent, 85% inhibition [95% CI: 76–92; n = 10] of TREK-2 channels, which was similar to that seen for TREK-1. Interestingly, for TREK-2, we observed a large over-recovery of current following compound washout to 201% above baseline [95% CI: 126–337; n = 7]. This over-recovery of current is similar to that reported previously [5], following sipatrigine block of TREK-1 channels. TREK-2 (and TREK-1) channels undergo alternative translation initiation (ATI) in their N terminal domains resulting in the expression of three functionally different isoforms of TREK-2 channels [[24], [25], [26], [27], [28], [29]]. We investigated the effect of sipatrigine on the three ATI-variants of TREK-2. Only the shortest form of the channel (TREK-2 Δ1-63) demonstrated significant alteration in drug response (p < 0.0001) with sipatrigine inhibition reduced to 51% [95% CI: 8–84; n = 6]. These data show that the distal half of the N terminus is necessary to promote full inhibition of TREK-2 by sipatrigine. This is similar to results seen with carvedilol inhibition as deletion of the N terminus in TREK-2 significantly decreased this compound's sensitivity [29]. A similar effect has also been shown on the short form of TREK-1 (Δ1-52) which showed less sensitivity to inhibition by both fluoxetine and pranlukast than WT TREK-1 channels, indicating that the N terminus of TREK-1 is involved in regulation of the channel [21,28].

Over recovery from inhibition was also lost in the shortest isoform of TREK-2 suggesting that the N terminus of the channel is also important for recovery of channel activity following application of sipatrigine. In TREK-1 channels, the N terminus interacts with the protein EBP50 in the plasma membrane which stabilises the structure of proteins and leads to a gradual run-up of current in the whole-cell configuration [30]. Therefore, loss of this interaction in the short form of TREK channels, may lead to a change in compound effects on the channel.

Lamotrigine (100 μM) had a small inhibitory effect on TREK-2 channels (14% [95% CI: 7–21; n = 10]). This was significantly smaller (p < 0.05) than that observed for lamotrigine block of TREK-1 and TRESK.

### 3.2 Disruption of a known contact drug binding site in the TM4 region of TREK-1 (L289A) reduces the magnitude of sipatrigine inhibition

**Fig. 2:**
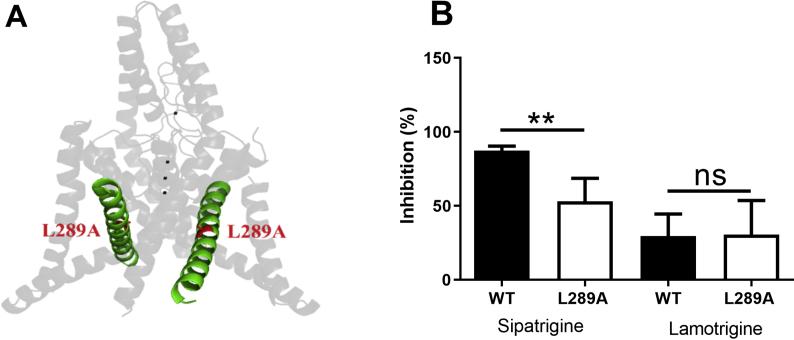
Effect of sipatrigine and lamotrigine on mutated TREK-1_L289A. (A) Homology model of human channel TREK-1 (based on PDB: 4TWK) highlighting location of mutated residue L289A (red) and TM4 region in green. (B) TREK-1 WT inhibition compared to TREK-1 L289A with sipatrigine and lamotrigine at a concentration of 100 μM (**p < 0.01). (For interpretation of the references to colour in this figure legend, the reader is referred to the Web version of this article.)

The co-crystallised structure of TREK-2 with norfluoxetine identified a number of amino acids important for compound binding [17] and showed that the residue leucine (L) at position 320 in the TM4 region of the channel is important for the binding and the inhibitory effect of norfluoxetine. The equivalent amino acid on TREK-1 (L289) (Fig. 2A) has recently been shown to contribute to the binding site for the TREK-1 activator BL-1249 and mutation of this residue reduces the effectiveness of the compound [31]. To determine if these sites were also important for sipatrigine and lamotrigine binding, we mutated L289 on TREK-1 and L320 on TREK-2 to an alanine (A). The inhibitory effect of sipatrigine (100 μM) on the TREK-1 L289A mutated current was significantly attenuated (56% [95% CI: 47–80; n = 9]), compared to the WT (87% [95% CI: 85–92; n = 19], p < 0.01). However, the inhibitory effect of lamotrigine (100 μM) remained unaffected by the mutation (31% [95% CI: 9–67; n = 6]) compared to the WT (30% [95% CI: 12–53; n = 6], p > 0.05, Fig. 2B). The inhibitory effect of sipatrigine (100 μM) on the equivalent mutation in TREK-2 (L320A) was also significantly reduced (67% [95% CI: 58–71; n = 9]) compared to WT (85% [95% CI: 76–92; n = 10], p < 0.001).

Therefore, it is suggested that equivalent leucine residue in both TREK-1 and TREK-2 channels is a determinant of sipatrigine binding, however, the same mutation in TREK-1 showed no change in the degree of lamotrigine inhibition. Thus, there is a difference in the “contact points” required for binding of these compounds to TREK channels.

We also investigated the effect of sipatrigine (100 μM) on a known gain-of-function (GOF) mutation of TREK-1, E306A [32], which has been shown to affect the magnitude of fluoxetine inhibition [6]. Sipatrigine (100 μM) inhibition of the E306A GOF mutation was 64% [95% CI: 48–74; n = 7] which was significantly less (p < 0.001) compared to WT TREK-1 current inhibition. Previous studies have shown that E306A mutation reduced the fluoxetine inhibitory effect due to the channel coupling to the membrane bilayer and entering the open-up state [6, see also 17]. Taken together, these results show that specific amino acids in the TM4 region of TREK channels are important for compound activity.

In this study, we provide evidence of a similar mechanism of block by sipatrigine to that described for fluoxetine, since inhibition is reduced by mutations that alter the fluoxetine binding site in TREK channels (L289A, L320A) and by the GOF mutation (E306A), that reduced inhibition by fluoxetine [6]. Taken together these data suggest that, like norfluoxetine, sipatrigine binds preferentially to the fenestration site on the channel revealed by the M4 down state [17,33] and that block is gating-state dependent [6,34]. Since there is known to be more than one structurally distinct open state of TREK channels [34], one might speculate that the over recovery from block (see also [5]), when sipatrigine leaves the fenestration site, is due to the channel preferentially entering an open state with a high Po, which is influenced by the N terminus of the channel, before eventual re-equilibration to the resting level of channel activity seen before application of sipatrigine.

### 3.3 Mutations in the central cavity of TRESK attenuate sipatrigine and lamotrigine inhibition

**Fig. 3:**
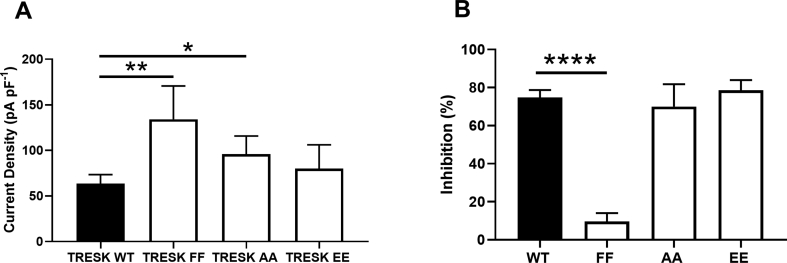
Effects of TRESK mutations on current density and sipatrigine inhibition. (A) Current density (pA pF^−1^) of WT and mutated TRESK channels (B) Sipatrigine (100 μM) inhibition of TRESK WT compared to TRESK FF, TRESK AA and TRESK EE (*p < 0.05, **p < 0.01, ****p < 0.0001).

For TRESK channels, the presence of bulky, phenylalanine (F) residues, F145 and F352, named the TM2.6 and TM4.6 residues, is unique for mammalian K2P channels [35,36]. It is suggested that these amino acids are exposed to a cytoplasmic vestibule in the inner pore region of the channel and that a mutation to a less bulky residue, such alanine, will diminish this exposure [18]. In agreement with our previous work [35] mutation of both these residues (F145A_F352A) results in a significantly increased current density (134 pA pF^−1^ [95% CI: 81–205; n = 13]), compared to WT (65 pA pF^−1^ [95% CI: 51–78; n = 28]) (Fig. 3A) (p < 0.01). This double F mutation was also found to significantly attenuate (p < 0.0001) the inhibitory effects of both sipatrigine (10% [95% CI: 4–17; n = 9]) and lamotrigine (5% [95% CI: 2 – 11; n = 11]) at a concentration of 100 μM (Fig. 3B).

These amino acids on TRESK channels have been suggested to be potential binding sites for channel blockers quinine, propafenone, lidocaine and loratadine [12,18]. However, the increase in basal current, [see also 35, 36] and the wide variety of structurally distinct molecules affected by mutation of these residues, suggests it is more likely that these mutations alter channel gating and indirectly occlude compound action rather than directly blocking the binding of the compounds.

### 3.4 The phosphorylation state of TRESK channel does not influence sipatrigine sensitivity

We considered whether sipatrigine inhibition of the TRESK channel was phosphorylation state dependent. The serine (S) residues (S252 and S264) have been identified as important sites for phosphorylation/dephosphorylation dependent regulation of TRESK channels [37]. Substitution of these residues to alanine alters the channel to mimic a “dephosphorylated” state, as the serine residues now cannot be phosphorylated. Alternatively, substituting these serine residues to a negatively charged glutamic acid (E) is thought to mimic a permanent “phosphorylated” channel state [37]. The “dephosphorylated” TRESK channel S252A_S264A (AA) had a significantly larger (p < 0.05) current density (95 pA pF^−1^ [95% CI: 68–122; n = 14]) than the WT channel (65 pA pF^−1^ [95% CI: 51–78; n = 28]). Whilst the phosphorylated S252E_S264E (EE) fixed channel had a similar (p > 0.05) current density to WT (80 pA pF^−1^ [95% CI: 49–104, n = 15]) (Fig. 3A). The inhibition by sipatrigine (100 μM) of the two fixed states were similar (p > 0.05) to that for TRESK WT with an inhibition of 70% [95% CI: 54–81; n = 6] for the TRESK AA channel and 79% [95% CI: 72–84; n = 6] for TRESK EE channel (see Fig. 3B).

### 3.5 Characterisation of CEN-092 on TRESK and TREK-1

**Fig. 4:**
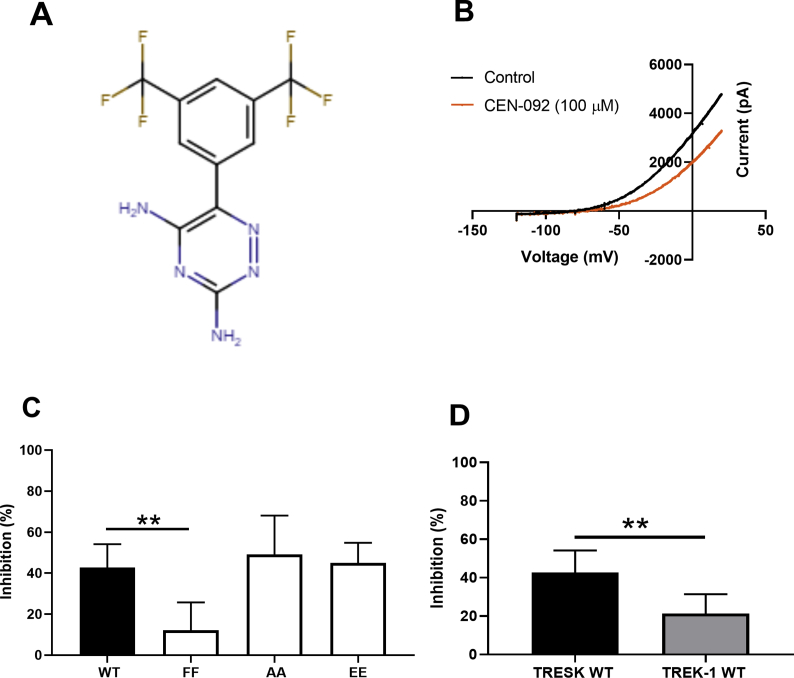
Inhibition of TRESK and TREK-1 channels by CEN-092. (A) Chemical structure of CEN-092. (B) Current-voltage relationship for TRESK channels in the presence and absence of CEN-092 obtained using the voltage-ramp protocol. (C) CEN-092 (100 μM) inhibition of WT and mutated TRESK channels. (D) Comparison between TRESK and TREK-1 channel inhibition by CEN-092 (100 μM) (**p < 0.01).

CEN-092 is one of a group of compounds developed as sodium channel blockers and shown, more recently, to be effective inhibitors of IFN gamma secretion [38]. CEN-092 (100 μM), which is derived from lamotrigine (Fig. 4A), inhibited TRESK current by 43% [95% CI: 31–54; n = 9], similar to the inhibition seen with lamotrigine. Furthermore, the TRESK FF mutated channel, significantly attenuated (p < 0.01) the magnitude of effect of CEN-092 (12% [95 CI: 1–33; n = 6]). The degree of inhibition by CEN-092 showed no significant difference (p > 0.05), between the WT and the two phosphorylated states of the channel (TRESK AA 49% [95% CI: 21–65; n = 6] and TRESK EE 45% [95% CI: 31–65; n = 8]), similar to that observed for sipatrigine inhibition (Fig. 4C). Inhibition of TREK-1 current by CEN-092 (100 μM) was 21% [95% CI: 5–30; n = 9), which was significantly lower than inhibition of TRESK at the same concentration (p < 0.01) (Fig. 4D).

Taken together, sipatrigine is the most potent of the three compounds and shows a small degree of selectivity for TREK channels over TRESK. Lamotrigine and the novel compound CEN-092, on the other hand, are less effective inhibitors of all three channels tested, but CEN-092 is significantly more effective at inhibiting TRESK than TREK channels. Given the paucity of selective antagonists of K2P channels [4], this study helps clarify the action of sipatrigine and lamotrigine on TREK and TRESK channels and, through, the comparative selectivity for the novel blocking agent CEN-092 for TRESK over TREK channels provides the potential for the development of more selective blocking agents in the future.

The putative importance of TREK and TRESK channels in pain signalling, suggests that pharmacological modulation which upregulates these channels may be a novel strategy for analgesia [4,39,40]. However, there are known analgesics which inhibit TRESK channels including acetaminophen, ibuprofen and nabumetone [41]. The mean plasma level of lamotrigine, which is currently used for trigeminal pain, in patients was 23 μM (5.9 mg/ml) with an upper level of 70 μM (18.1 mg/ml) [42] which would cause significant block of both TRESK and TREK channels in these patients.

Analgesics from traditional medicine such as aristolochic acid and hydroxyl-α-sanshool, the active ingredient of Szechuan peppers, have also been shown to block TRESK channels [19]. The effect of hydroxyl-α-sanshool is believed to be caused by desensitising the excitatory neurons which leads to a numbing sensation. Thus, it appears that both activation and inhibition of TREK and TRESK channels may be beneficial in the treatment of pain and there is, perhaps, an optimal level of channel activity required to regulate nociceptive neuron excitability.

There is also significant research which suggests that TREK-1 channels are involved in depression and a potential target for antidepressants. Deletion of the TREK-1 gene (KCNK2) resulted in a depression-resistant phenotype in mice [7]. Several antidepressants are known to inhibit TREK-1 channels including fluoxetine and citalopram [9,43]. Lamotrigine is already used in patients with depression linked to bipolar disorder and has reportedly been used in treatment for patients resistant to first line antidepressants. Since sipatrigine is a more potent TREK-1 channel antagonist than lamotrigine, it could be a more effective agent for treating specific forms of depression [44].

## Declaration of competing interest

The authors declare the following financial interests/personal relationships which may be considered as potential competing interests:

L. Harbige, M. Leach, Triazine derivatives as interferon-gamma inhibitors, US Patent Application Publication. US (2018) 0169105 A1.

Related research on K2P channels is supported by a Centre for Therapeutic Discovery Award from LifeArc to A Mathie and E Veale.
